# Cell Selection Technique for Millimeter-Wave Cellular Systems with Hybrid Beamforming

**DOI:** 10.3390/s17061461

**Published:** 2017-06-21

**Authors:** Mohammed Saquib Khan, Sung Joon Maeng, Yong Soo Cho

**Affiliations:** School of Electrical and Electronic Engineering, Chung-Ang University, Seoul 156-756, Korea; snhk02@gmail.com (M.S.K.); aod0527@naver.com (S.J.M.)

**Keywords:** millimeter-wave, cell selection, hybrid beamforming, channel estimation

## Abstract

In this paper, a cell selection technique for millimeter-wave (mm-wave) cellular systems with hybrid beamforming is proposed. To select a serving cell, taking into account hybrid beamforming structures in a mm-wave cellular system, the angles of arrival and departure for all candidate cells need to be estimated in the initialization stage, requiring a long processing time. To enable simultaneous multi-beam transmissions in a multi-cell environment, a cell and beam synchronization signal (CBSS) is proposed to carry beam IDs in conjunction with cell IDs. A serving cell maximizing the channel capacity of the hybrid beamformer is selected with the estimated channel information and the optimum precoder. The performance of the proposed technique is evaluated by a computer simulation with a spatial channel model in a simple model of a mm-wave cellular system. It is shown by simulation that the proposed technique with the CBSS can significantly reduce the processing time for channel estimation and cell selection, and can achieve additional gains in channel capacity, or in bit error rate, compared to that obtained by conventional techniques.

## 1. Introduction

Owing to the wide use of smartphones and sensor nodes for various applications and services, mobile data traffic is growing rapidly. In particular, sensor nodes such as CMOS sensors and charge-coupled device (CCD) cameras require a high data rate transmission. Very large arrays and/or higher frequency spectrum are recognized to accommodate the increase in mobile traffic. Massive multiple-input multiple-output (MIMO) is expected to bring large gains in terms of spectral and energy efficiency and robustness to hardware failures and impairments [1,2,3]. Further, millimeter wave (mm-wave) communication is a promising technology that allows the use of a wide bandwidth for the high data rates required for next-generation outdoor cellular systems [4,5]. However, mm-wave signals experience severe large-scale path losses compared to lower-frequency band signals [5,6]. To compensate for the large path losses, and to increase the transmission range in the mm-wave frequency band, highly directional beamforming antennas are necessary at both the base station (BS) and the mobile station (MS). Fortunately, a decrease in wavelength enables a large number of antenna arrays to be packed into small form factors, making it feasible to realize the large arrays needed for high beamforming gains [7,8].

Traditionally, MIMO processing is often performed digitally in baseband to support multi-stream service in multipath environments. To perform digital beamforming at mm-wave frequencies, each antenna element requires dedicated baseband and radio frequency (RF) hardware, resulting in high costs and a substantial amount of power consumption. This has constrained mm-wave systems to rely heavily on analog or RF processing [8,9]. Analog beamforming has hitherto been used in the mm-wave systems; however, it is suboptimal compared to digital beamforming. As analog beamforming relies entirely on RF domain processing, the performance is limited by quantization of phase angles and the support of only single stream transmissions [10,11]. In order to overcome the issues faced by digital and analog beamforming, an alternative beamforming architecture, known as hybrid beamforming, has been introduced [12,13]. In hybrid beamforming, beamforming is partitioned into analog and digital domains. Because of the additional digital processing, more degrees of freedom are available in designing the hybrid precoder compared to analog-only beamformers. In addition, hybrid beamforming allows for the supporting of multi-stream and multi-user transmissions.

However, in mm-wave systems, analog beamforming has been used to date for beam training and cell selection in the initialization stage. In the standards of wireless LAN and wireless PAN (IEEE 802.11ad and IEEE 802.15c) widely used for wireless sensor networks (WSNs), analog beamforming is used for both beam training and data transmission using the 60 GHz unlicensed spectrum [14,15,16,17]. In the pre-5G specification for 5G mm-wave cellular systems, called KT Pyeongchang 5G Technical Specification (5G-SIG), analog beamforming is used for beam training and cell selection in the initialization stage. However, both analog beamforming and hybrid beamforming are allowed in the data transmission stage [18,19]. The 5G-SIG, approved by leading world companies in wireless communication, was released in 2016 to demonstrate 5G pilot services for the Pyeongchang Winter Olympic Games in February 2018. In 5G-SIG directional beamforming, antennas are used to maintain the link budget during the initialization stage. The serving cell is found by time-division beam switching (TDBS) in which transmit (Tx) beams of candidate BSs, and receive (Rx) beams of MS, are swept to measure signal-to-noise ratios (SNRs) for all possible links. The serving cell and Tx–Rx beam pair with the highest SNR in the initialization stage are selected. However, the conventional cell selection technique, in which the serving cell is selected based on the measurements of Tx–Rx beam pairs for all possible links (candidate cells), may not be optimal in multipath environments. For example, in a multipath channel where one line-of-sight (LoS) and multiple non-line-of-sight (NLoS) paths are available in candidate cells, only a single path providing the highest SNR (possibly LoS) will be used to select the serving cell in the conventional technique. However, the selected cell in the initialization stage may not be optimal in the data transmission stage where hybrid beamforming is employed. So far, to the best of our knowledge, there has been no attempt to select a serving cell in the initialization stage by considering the hybrid beamforming structure to be used in the data transmission stage. 

In this paper, a cell selection technique for mm-wave cellular systems in the initialization stage is proposed for hybrid beamforming structures in data transmission stage. To select a serving cell, taking into consideration the hybrid beamforming structure in the initialization stage, we need to estimate angles of arrival (AoA) and angles of departure (AoD) for all candidate cells. The processing time required for channel estimation increases proportionally to the product of the number of Tx beams, the number of Rx beams, and the number of candidate cells [20]. This long processing time will create significant overheads for a moving MS because the channel estimation should be performed periodically for a possible handover. A new channel estimation technique is proposed by transmitting multiple beams simultaneously, where each beam is mapped with a unique proposed cell and beam synchronization signal (CBSS), all in the physical layer. In the proposed CBSS, the beam ID (BID) is designed in conjunction with cell ID (CID), because the BID must be detected in a multicell environment. It is shown that multiple beams with different BIDs can be transmitted simultaneously with minimal inter-beam interference in a multicell environment, resulting in a significant reduction in processing time for channel estimation and cell selection. The hierarchical structure of CBSS gives the advantage of reusing the same BIDs in adjacent cells, allowing us to generate a large set of sequences. In the proposed cell selection technique, the serving cell is selected based on the maximum channel capacity of the hybrid beamformer with the estimated channel information and optimum precoder. Moreover, the serving cell and the corresponding AoDs and AoAs are determined by taking into consideration the number of RF chains that will be used in the data transmission stage.

The remainder of the paper is organized as follows. Section 2 summarizes the conventional cell selection technique for mm-wave cellular systems and the system model that will be used in this paper. In Section 3, the cell selection technique for mm-wave cellular systems with hybrid beamforming is proposed, after describing the properties of CBSS and channel estimation technique using the CBSS. In Section 4, the performance of the proposed technique is verified by computer simulation using a simple model of a mm-wave cellular system. Finally, conclusions are drawn in Section 5. The following notations are used throughout the paper: A is a matrix, a is a vector, and A is set. ${\left[\cdot \right]}^{*}$, ${\left(\cdot \right)}^{\prime }$, ⌊⋅⌋, ⌈⋅⌉, and < > are conjugate, conjugate transpose, floor operation, ceiling operation, and Jacobi symbol, respectively. $N_{TX}$ and $N_{B}$ denote the number of antennas and beams at the BS while $N_{RX}$ and $N_{I}$ denote the number of antennas and beams at the MS, respectively. $N_{S}$ is the number of data streams and $N_{RF}$ is the number of RF transmit chains. *c* is the cell ID, $\bar{c}$ is reference cell ID, *b* is the beam ID, and $\bar{b}$ is reference beam ID. I is the identity matrix and $1_{N}$ is the *N*-length all-ones vector. A⊗B is the Kronecker product of **A** and **B**. 

## 2. Preliminaries 

### 2.1. Conventional Cell Selection Technique

A cell search procedure that will be used for an MS in conventional LTE systems can be summarized as follows. The MS first needs to scan by measuring the received signal strength indicators (RSSIs) of all supported carriers for entire bandwidth, each with 20 MHz. The measured RSSIs include the signal strength of the desired cell, interference cells, and noise. Then, RSSIs lower than the predefined threshold (or signal strength sensitivity) are filtered, resulting in a list of surviving candidates. Each surviving candidate is processed by cell identification block until the MS finds a cell on which to camp. Cell identification involves primary synchronization signal (PSS) and secondary synchronization signal (SSS) detection. The SSS carries the information of cell ID group, while the PSS carries the information of cell ID within the cell ID group. A physical cell ID is constructed by combining the cell ID group and cell ID within the cell ID group after PSS and SSS detection.

However, in mm-wave cellular systems, the selection of the best beam pair, as well as the cell selection, is important because a maximum array gain is obtained in these systems when Tx and Rx beams are perfectly aligned [14,15]. Especially for systems with narrow beams, a small mismatch or misalignment between Tx and Rx beams may result in a substantial loss in the received power. In mm-wave cellular systems, the TDBS scheme has been used to find the serving cell with the best beam pair in the initialization and handover stages [18,19]. In the TDBS scheme, individual Tx beams are transmitted from the BS until all the Tx beams are transmitted. The Rx beam sweep is performed at the MS for each Tx beam to measure the SNR for each Tx-Rx beam pair. The measurement of the SNRs for all possible Tx-Rx beam pairs must be performed for all candidate cells to select a serving BS with the best beam pair. Therefore, the serving cell and best beam pair are selected based on the measurement results obtained by all possible Tx-Rx beam pairs of candidate cells. A physical cell ID of the serving cell is obtained by detecting the PSS and SSS transmitted from the serving cell. A beam pair with maximum SNR is selected as the best beam pair and the corresponding cell with the physical cell ID as the serving cell. However, the selected cell and beam pair may not be optimal for hybrid beamforming system because the effect of the multipath channel is not considered in the initialization stage [10,11,14]. 

**Figure 1:**
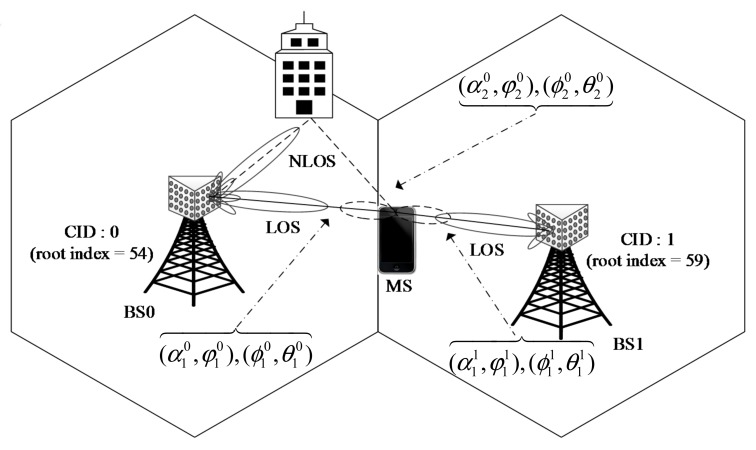
Example of a mm-wave cellular system with two BSs.

Figure 1 shows an example of a mm-wave cellular system with two BSs with different CIDs. Here, it is assumed that a multipath (NLoS) component exists in cell 0 and an MS is located at the cell boundary. It is also assumed that the LoS path is stronger than the NLoS path in cell 0. Here, switched beamforming is used at both the BSs and the MS. In this example, a serving cell will be selected by measuring the signal strengths of LoS paths from two cells. The cell with the higher signal strength of the LoS path will be selected [18,19]. Here, the multipath (NLoS) component is not used for cell selection. However, in hybrid beamforming systems, the multipath channel is used to increase the SNR in power-limited conditions (e.g., cell edge), or to increase user data rates, as long as the multipath channel has sufficiently different propagation paths. Therefore, the serving cell selected in the initialization stage may not be optimal for hybrid beamforming system in the data transmission stage.

### 2.2. System Model

Consider a mm-wave cellular system in which a transmitter with $N_{TX}$ antennas transmitting $N_{S}$ data streams to a receiver with $N_{RX}$ antennas. It is assumed that, in the data transmission stage, hybrid beamforming is used at the BS, and analog beamforming with a set of predefined angles is used at the MS. The BS is equipped with $N_{RF}$ transmit chains to enable $N_{S}$ data streams transmission, such that $N_{S}\leq N_{RF}\leq N_{TX}$ [12,21]. This architecture enables the transmitter to apply an $N_{RF}\times N_{S}$ baseband precoder by using $N_{RF}$ transmit chains, followed by an $N_{TX}\times N_{RF}$ RF precoder using analog circuitry. In the proposed technique, it is assumed that all Tx beams are simultaneously transmitted from all candidate BSs, and Rx beams are swept over in this period, to reduce the processing time required for channel estimation. All Tx beams are transmitted repeatedly until one round of Rx beam sweep is completed. In the proposed technique, CBSSs are mapped to the corresponding Tx beams, simultaneously transmitted from candidate BSs. Because the BID needs to be detected in a multicell environment with multiple beams, the CBSS is designed such that the BID is mapped on a synchronization sequence in association with its CID. Both the BID and CID are transmitted on a single CBSS to facilitate joint detection of the BID and CID. This hierarchical structure enables the MS to identify the CID of the beam from the received CBSS. In a mm-wave cellular system based on orthogonal frequency division multiplexing (OFDM), the signals received by the *i*-th Rx beam at the MS in the initialization stage can be expressed in the frequency domain as follows:

(1) $$Y_{i}\left(k\right)=\sum_{c=0}^{N_{C}-1}\sum_{b=0}^{N_{B}-1}{q_{i}}^{\prime }H^{c}\left(k\right)p^{c,b}X^{c,b}\left(k\right)+{q_{i}}^{\prime }W\left(k\right)$$

where $q_{i}\in {\mathbb{C}}^{N_{RX}\times 1}$ and $p^{c,b}\in {\mathbb{C}}^{N_{TX}\times 1}$ denote an analog combiner for the *i*-th Rx beam at the MS, and an analog precoder for the *b*-th beam in the *c*-th cell, respectively. Here, $N_{I}$, $N_{B}$, and $N_{C}$ denote the number of Rx beams at the MS, the number of Tx beams at the BS, and the number of candidate cells, respectively. $H^{c}\left(k\right)$ is an $N_{RX}\times N_{TX}$ matrix at subcarrier *k* that represents the mm-wave channel between the $N_{B}$ beams of the *c*-th cell and the $N_{I}$ Rx beams of the MS. $X^{c,b}\left(k\right)$ and W(k) denote the CBSS transmitted from the *b*-th beam of the *c*-th cell and additive white Gaussian noise (AWGN), respectively. If a total number of Tx beams at the BS is $N_{B}$ and the number of available RF transmit chains is $N_{RF}(\leq N_{B})$, then only $N_{RF}$ beams can be simultaneously transmitted, and $N_{I}$ Rx beams are swept over in this period. The same procedure needs to be repeated $N_{B}/N_{RF}$ times by switching the set of $N_{RF}$ beams until all the Tx beams are transmitted. For example, if the values of $N_{B}$, $N_{RF}$, and $N_{I}$ are 16, 1, and 8, respectively, then only one beam will be transmitted until one round of Rx beam sweep is completed. This procedure will be repeated 16 times. If the value of $N_{RF}$ is 8, then 8 beams will be simultaneously transmitted until one round of Rx beam sweep is completed. This procedure will be repeated only 2 times. Thus, the processing time required for channel estimation increases as the number of RF transmit chains decreases. In this paper, it is assumed that the number of RF transmit chains is the same as the number of Tx beams for notational convenience.

In Equation (1), the matrix $H^{c}\left(k\right)$ represents the channel at subcarrier *k*, which is derived from the delay-τ channel matrix $H^{c}\left(\tau \right)$. Here, the delay-τ channel matrix can be considered as a geometric wideband channel model, which can be viewed as the sum of $N_{M}$ scattering clusters each with time delay $\tau_{m}$. Each cluster consists of $N_{L}$ propagation rays with relative time delay $\tau_{l}$. The delay-τ channel matrix $H^{c}\left(\tau \right)$ can be written as [22]:

(2) $$H^{c}\left(\tau \right)=\sqrt{\frac{N_{TX}N_{RX}}{N_{M}N_{L}}}\sum_{m=1}^{N_{M}}\sum_{l=1}^{N_{L}}\beta_{m,l}^{c}p_{rc}\left(dT_{S}-\tau_{m}-\tau_{l}\right)a_{RX}\left(\alpha_{ml}^{c},\varphi_{ml}^{c}\right){a_{TX}}^{\prime }\left(\phi_{ml}^{c},\theta_{ml}^{c}\right)$$

and the channel at subcarrier *k*, $H^{c}\left(k\right)$, can be expressed as [23]:

(3) $$H^{c}\left(k\right)=\sum_{\tau =0}^{N_{\tau }-1}H^{c}\left(\tau \right)e^{\frac{-j2\pi k}{N_{z}}\tau }$$

where $N_{L}$ and $N_{M}$ denote the number of propagation rays and the number of clusters, respectively. $p_{rc}\left(\tau \right)$ is the pulse shaping function for $T_{S}$-spaced signaling evaluated at τ second, $\beta_{m,l}^{c}$ is the complex gain of the *l*-th ray in the *m*-th cluster, whereas $a_{RX}\left(\alpha_{ml}^{c},\varphi_{ml}^{c}\right)$ and $a_{TX}\left(\phi_{ml}^{c},\theta_{ml}^{c}\right)$ represent the Rx and Tx array response vectors at an azimuth (elevation) angle of $\alpha_{ml}^{c}\left(\varphi_{ml}^{c}\right)$ and $\phi_{ml}^{c}\left(\theta_{ml}^{c}\right)$, respectively. The array response vector for the *N*-element uniform linear array (ULA) on the *y*-axis and uniform planar array (UPA) in the *xy*-plane with $N_{e}$ and $N_{f}$ elements on the *x* and *y* axes, respectively, is given by [24]:

(4) $$a_{TX}^{\mathrm{ULA}}\left(\phi \right)=\frac{1}{\sqrt{N}}{\left[1,\;e^{j\frac{2\pi }{\lambda }d\sin\left(\phi \right)},\;\ldots ,\;e^{j\frac{2\pi }{\lambda }d\left(N-1\right)\sin\left(\phi \right)}\right]}^{T}$$

(5) $$\begin{array}{cc} a_{TX}^{\mathrm{UPA}}\left(\phi ,\theta \right)= & \frac{1}{\sqrt{N_{e}N_{f}}}[1,\;\ldots ,\;e^{j\frac{2\pi }{\lambda }d\left(e\cos\left(\phi \right)\sin\left(\theta \right)+f\sin\left(\phi \right)\sin\left(\theta \right)\right)},\\ & \ldots ,\;e^{j\frac{2\pi }{\lambda }d\left(\left(N_{e}-1\right)\cos\left(\phi \right)\sin\left(\theta \right)+\left(N_{f}-1\right)\sin\left(\phi \right)\sin\left(\theta \right)\right)}{]}^{T} \end{array}$$

where *d* is the element spacing and λ is the wavelength. Also, $0\leq e<N_{e}$ and $0\leq f<N_{f}$ are antenna element indices for *x* and *y*, respectively.

## 3. Proposed Cell Selection Technique 

### 3.1. Cell and Beam Synchronization Signal (CBSS)

**Figure 2:**
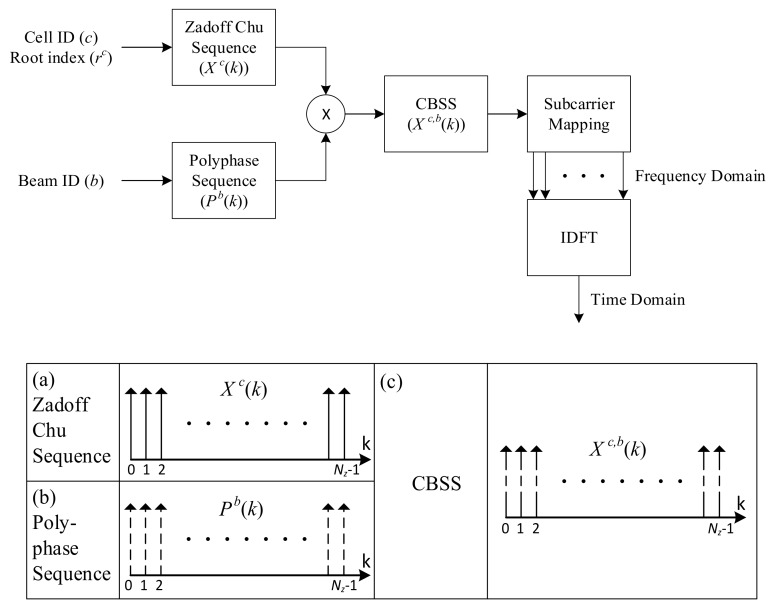
The generation process of CBSS.

In order to select a serving cell, we need to estimate channels (AoAs and AoDs) for all candidate cells in the initialization stage. In the proposed technique, CBSSs are simultaneously transmitted from all Tx beams of candidate BSs to reduce the processing time required for channel estimation. Here, it is assumed that the synchronization and CID detection are performed by the conventional primary synchronization signal (PSS) and secondary synchronization signal (SSS), used in LTE systems [25]. In addition, it is assumed that one additional OFDM symbol carries the proposed CBSS, next to the SSS. The CBSS needs to carry the CID, as well as the BID, in order to be detected in the multi-cell environment. If the CBSS carries only BID information, it will be impossible to determine which cell had transmitted the BID. Therefore, a large number of different sequences (CID × BID) need to be generated. In order to transmit the information in one OFDM symbol, the BID is mapped in association with the CID in the CBSS. The proposed CBSS can be viewed as a combination of a Chu sequence and a polyphase sequence [26]. In the proposed CBSS, a new sequence is generated by mapping the CID to the root index of the Chu sequence and the BID to the index of the polyphase sequence. Figure 2 shows the generation process of the CBSS. 

The CBSS is defined by the product of a prime-length Chu sequence $X^{c}\left(k\right)$ and a polyphase sequence $P^{b}\left(k\right)$ in the frequency domain as follows:

(6) $$\begin{array}{cc} X^{c,b}(k)= & X^{c}\left(k\right)P^{b}\left(k\right)\\ X^{c}\left(k\right)= & e^{\frac{j\pi r^{c}k(k+1)}{N_{z}}},\;P^{b}\left(k\right)=e^{\frac{-j2\pi bkL}{N_{z}}},\;k=0,1,\ldots ,N_{z}-1 \end{array}$$

where, $c\in N_{C}$, $b\in N_{B}$, $r^{c}\in N_{R}$, $N_{C}=\left\{\begin{array}{cccc} 0 & 1 & \ldots & N_{C}-1 \end{array}\right\}$, $N_{B}=\left\{\begin{array}{cccc} 0 & 1 & \ldots & N_{B}-1 \end{array}\right\}$, and $N_{R}=\left\{\begin{array}{cccc} 1 & 2 & \ldots & N_{R} \end{array}\right\}$ denote the CID, BID, Chu’s root index mapped with CID c, set of CIDs, set of BIDs, and set of all possible Chu’s root indices, respectively. $N_{R}$ and $N_{z}$ denote the number of all possible Chu’s root index and sequence length, respectively. In the polyphase sequence of CBSS, a parameter L is introduced to avoid the incorrect detection of cell ID when a symbol timing offset (STO) exists.

Next, the properties of the CBSS are analyzed. The relationship between the frequency domain and time domain of the CBSS is given by:

(7) $$\begin{array}{cc} x^{c,b}\left(n\right)= & \frac{1}{N}\sum_{k=0}^{N_{z}-1}X^{c,b}\left(k\right)e^{\frac{j2\pi kn}{N_{z}}}\\ & =\frac{1}{N}\sum_{k=0}^{N_{z}-1}e^{\frac{j\pi r^{c}}{N_{z}}\left(k(k+1)-2bkL{\left(r^{c}\right)}^{-1}+2kn{\left(r^{c}\right)}^{-1}\right)}\\ & =e^{\frac{-j\pi }{N_{z}}\left({\left(r^{c}\right)}^{-1}{\left(n-bL\right)}^{2}+\left(n-bL\right)\right)}\times \frac{1}{N}\sum_{k=0}^{N_{z}-1}X\left(k+{\left(r^{c}\right)}^{-1}\left(n-bL\right)\right)\\ ∴x^{c,b}\left(n\right) & =x^{c}\left(0\right)X^{c^{*}}\left({\left(r^{c}\right)}^{-1}\left(n-bL\right)\right) \end{array}$$

where $x^{c}\left(0\right)=\frac{1}{\sqrt{N_{z}}}\langle \frac{\chi r^{c}}{N_{z}}\rangle \frac{1-j^{N_{z}}}{1-j}e^{\frac{-j2\pi }{N_{z}}r^{c}\chi \gamma^{2}}$, $\chi =\frac{N_{z}+1}{2},\;\gamma =\frac{N_{z}-1}{2}$.

The autocorrelation function of the CBSS in the frequency domain is given by:

(8) $$\begin{array}{cc} R^{c,b}\left(l\right) & =\sum_{k=0}^{N_{z}-1}X^{c,b}\left(k\right){\left[X^{c,b}\left(k+l\right)\right]}^{*}\\ & =\sum_{k=0}^{N_{z}-1}e^{\frac{j\pi }{N_{z}}\left(r^{c}l\left(l+1\right)-2Lbl\right)}e^{\frac{j2\pi }{N_{z}}\left(r^{c}kl\right)}\\ & =N_{z}e^{\frac{j\pi }{N_{z}}\left(r^{c}l\left(l+1\right)-2Lbl\right)}\delta \left(\mathrm{mod}\left(l,N_{z}\right)\right) \end{array}$$

where δ(⋅), ${\left(r^{c}\right)}^{-1}$, and $\langle \chi r^{c}/N_{z}\rangle$ denote the Kronecker delta function, the multiplicative inverse of $r^{c}$, and the Legendre symbol with a value of −1, 0, or 1, respectively. As seen in Equation (7), the time domain version of the CBSS can be represented as a Chu sequence with a cyclic shift and its amplitude is given by $\left|x^{c,b}\left(n\right)\right|=1/\sqrt{N_{z}}$ for all *n*. From Equations (7) and (8), it can be seen that the time domain version of CBSS, $x^{c,b}\left(n\right)$, has a constant amplitude and zero-autocorrelation except at the lag *l* where mod(l,N)=0, thereby satisfying the constant-amplitude zero-autocorrelation (CAZAC) property. The CAZAC property holds if and only if Chu’s root index and sequence length are co-prime. The intra-cell correlation (correlation between two beams b and $\bar{b}$ in the same cell) and inter-cell correlation (correlation between a beam in one cell and a beam in the adjacent cell) are analyzed in an ideal condition, ignoring the channel and noise effects, as follows:

(9) $$\begin{array}{cc} Z^{c,\bar{b}} & =\sum_{k=0}^{N_{z}-1}X^{c,b}(k){\left[X^{c,\bar{b}}(k)\right]}^{*}\\ & =\sum_{k=0}^{N_{z}-1}e^{\frac{j\pi }{N_{z}}\left(r^{c}k(k+1)-2bLk-r^{c}k(k+1)+2\bar{b}Lk\right)}\\ & =\sum_{k=0}^{N_{z}-1}e^{\frac{j2\pi }{N_{z}}kL\left(\bar{b}-b\right)}=N_{z}\delta \left(\mathrm{mod}\left(b,\bar{b}\right)\right) \end{array}$$

and

(10) $$\begin{array}{cc} Z^{\bar{c},\bar{b}} & =\sum_{k=0}^{N_{z}-1}X^{c,b}(k){\left[X^{\bar{c},\bar{b}}(k)\right]}^{*}\\ & =\sum_{k=0}^{N_{z}-1}e^{\frac{j\pi }{N_{z}}\left(r^{c}k(k+1)-2bLk-r^{\bar{c}}k(k+1)+2\bar{b}Lk\right)}\\ & =e^{\frac{-j\pi }{N_{z}}L\left(\bar{b}-b\right)\left({\left(r^{c}-r^{\bar{c}}\right)}^{-1}L\left(\bar{b}-b\right)+1\right)}\sum_{k=0}^{N_{z}-1}e^{\frac{j\pi }{N_{z}}k(k+1)\left(r^{c}-r^{\bar{c}}\right)}\\ & =e^{\frac{-j\pi }{N_{z}}L\left(\bar{b}-b\right)\left({\left(r^{c}-r^{\bar{c}}\right)}^{-1}L\left(\bar{b}-b\right)+1\right)}\sum^{c,\bar{c}} \end{array}$$

where

(11) $$\sum^{c,\bar{c}}=\sqrt{N_{z}}\left\{ \begin{array}{l} \langle \frac{\chi \left|r^{c}-r^{\bar{c}}\right|}{N_{z}}\rangle \frac{1-j^{N_{z}}}{1-j}e^{\frac{-j2\pi }{N_{z}}\left(r^{c}-r^{\bar{c}}\right)\chi \gamma^{2}},\;r^{c}>r^{\bar{c}}\\ \langle \frac{\chi \left|r^{c}-r^{\bar{c}}\right|}{N_{z}}\rangle \frac{1+j^{N_{z}}}{1+j}e^{\frac{-j2\pi }{N_{z}}\left(r^{c}-r^{\bar{c}}\right)\chi \gamma^{2}},\;r^{c}<r^{\bar{c}} \end{array}\right.$$

Equation (10) can be derived using the Gauss sum property [27]. From Equations (9) and (10), it can be observed that the intra-cell correlation has a peak amplitude $N_{z}$ only when $b=\bar{b}$ for $b,\bar{b}\in N_{B}$, and the amplitude of the inter-cell correlation is given by $\sqrt{N_{z}}$. As can be seen in Equations (9) and (10), CBSSs with the same Chu’s root indices are perfectly orthogonal, and CBSSs with different Chu’s root indices are not orthogonal. However, the interference term caused by the beam transmitted from neighboring BSs is relatively small compared to the signal from the reference beam. For example, the ratio between $\sqrt{N_{z}}$ and $N_{z}$ is 3% when $N_{z}=1021$. When an STO ($\delta_{t}$) is present, the intra-cell correlation is given by:

(12) $$\begin{array}{cc} Z^{c,\bar{b}} & =\sum_{k=0}^{N_{z}-1}X^{c,b}(k)e^{\frac{-j2\pi k\delta_{t}}{N_{z}}}{\left[X^{c,\bar{b}}(k)\right]}^{*}\\ & =\sum_{k=0}^{N_{z}-1}e^{\frac{j\pi }{N_{z}}\left(r^{c}k(k+1)-2bLk-r^{c}k(k+1)+2\bar{b}Lk-2k\delta_{t}\right)}\\ & =\sum_{k=0}^{N_{z}-1}e^{\frac{j2\pi }{N_{z}}k\left(L\left(\bar{b}-b\right)-\delta_{t}\right)}\\ & =N_{z}\delta \left(\mathrm{mod}\left(b-\bar{b},\delta_{t}\right)\right) \end{array}$$

The correlation value in Equation (12) becomes high when the following condition is satisfied:

(13) $$L=\frac{\delta_{t}}{\Delta b},\;L>0$$

where Δb is defined as $\bar{b}-b$. To avoid the condition in Equation (13), the parameter *L* needs to be adjusted and can be set to the following value:

(14) $$L>\delta_{t_{\max}},\;L_{\max}=\left\lfloor \frac{N_{z}}{N_{B}}\right\rfloor$$

where $\delta_{t_{\max}}$ and $L_{\max}$ denote the maximum STO and maximum value of parameter *L*, respectively. The value of *L* must increase proportionally with respect to the STO, because of the time delay between the BS and MS. This can result in the reduction of the number of BIDs to $\left\lfloor N_{z}/L\right\rfloor$.

### 3.2. Channel Estimation and Cell Selection

Using the CBSSs, channels of candidate cells are estimated in the initialization stage. The channel estimation begins with the correlation between the received signal in Equation (1) and the known CBSSs. The correlation value between the signal received from the *i*-th Rx beam at the MS and reference CBSS is given by:

(15) $$\begin{array}{cc} Z_{i}^{\bar{c},\bar{b}} & =\sum_{k=0}^{N_{z}-1}Y_{i}\left(k\right){\left[X^{\bar{c},\bar{b}}\left(k\right)\right]}^{*}\\ & =\sum_{k=0}^{N_{z}-1}\sum_{c=0}^{N_{c}-1}\sum_{b=0}^{N_{b}-1}{q_{i}}^{\prime }H^{c}\left(k\right)p^{c,b}X^{c,b}\left(k\right){\left[X^{\bar{c},\bar{b}}\left(k\right)\right]}^{*}+\sum_{k=0}^{N_{z}-1}{q_{i}}^{\prime }W\left(k\right){\left[X^{\bar{c},\bar{b}}\left(k\right)\right]}^{*}\\ & ={q_{i}}^{\prime }\left(\sum_{k=0}^{N_{z}-1}H^{\bar{c}}\left(k\right)X^{\bar{c},\bar{b}}\left(k\right){\left[X^{\bar{c},\bar{b}}\left(k\right)\right]}^{*}\right)p^{\bar{c},\bar{b}}\\ & +\sum_{b=0,b\neq \bar{b}}^{N_{b}-1}{q_{i}}^{\prime }\left(\sum_{k=0}^{N_{z}-1}H^{\bar{c}}\left(k\right)X^{\bar{c},b}\left(k\right){\left[X^{\bar{c},\bar{b}}\left(k\right)\right]}^{*}\right)p^{\bar{c},b}\\ & +\sum_{c=0,c\neq \bar{c}}^{N_{c}-1}\sum_{b=0}^{N_{b}-1}{q_{i}}^{\prime }\left(\sum_{k=0}^{N_{z}-1}H^{c}\left(k\right)X^{c,b}\left(k\right){\left[X^{\bar{c},\bar{b}}\left(k\right)\right]}^{*}\right)p^{c,b}+\sum_{k=0}^{N_{z}-1}{q_{i}}^{\prime }W\left(k\right){\left[X^{\bar{c},\bar{b}}\left(k\right)\right]}^{*} \end{array}$$

where $X^{\bar{c},\bar{b}}\left(k\right)$ denote the reference CBSS with the reference CID $\bar{c}$ and BID $\bar{b}$. The correlation in Equation (15) should be performed for $N_{C}$ cells and $N_{B}$ Tx beams. Here, the vector $q_{i}$ is constructed to form an *i*-th Rx beam in the direction of *m*-th cluster, thereby receiving the signals only from the *m*-th cluster. If the $N_{L}$ propagation rays contributed by the *m*th cluster are received within the sampling interval ($T_{S}$), the channel $H^{c}\left(k\right)$ with Tx and Rx beamforming can be written as $H^{\bar{c}}$. For example, when only two cells (cell 0 and cell 1) are present, the correlation in Equation (15) is given by:

(16) $$Z_{i}^{0,\bar{b}}=N_{z}\left({q_{i}}^{\prime }H^{0}p^{0,\bar{b}}\right)+0+\sqrt{N_{z}}\sum_{b=0}^{N_{b}-1}\left({q_{i}}^{\prime }H^{1}p^{1,b}\right)$$

where the reference CID, $\bar{c}$, is set to 0 and the noise effect is ignored. From the first two terms in Equations (15) and (16), it can be observed that there is no interference from other beams in the same cell. That is, the correlation value is high ($N_{z}$) when BIDs are the same, and the correlation value is zero when other BIDs are used. The third term in Equations (15) and (16) represents the interference term ($\sqrt{N_{z}}$) from beams in the other cells. In a vector form, ${q_{i}}^{\prime }H^{\bar{c}}p^{\bar{c},b}$ can also be written as follows:

(17) $$\begin{array}{cc} {q_{i}}^{\prime }H^{\bar{c}}p^{\bar{c},b} & =\left({\left(p^{\bar{c},b}\right)}^{T}⊗{q_{i}}^{\prime }\right)\mathrm{vec}\left(H^{\bar{c}}\right)\\ & =\left({\left(p^{\bar{c},b}\right)}^{T}⊗{q_{i}}^{\prime }\right)\left(A_{TXD}^{*}⊗A_{RXD}\right)\beta^{\bar{c}}=A^{\bar{c}}\beta^{\bar{c}} \end{array}$$

where $\beta^{\bar{c}}$ is unknown path gains of $N_{M}N_{L}$ propagation paths. The array response matrices (unitary discrete Fourier transform [DFT] matrices) at the BS and MS are defined for ULAs and UPAs, as follows:

(18) $$\begin{array}{c} A_{TXD}=\left\{ \begin{array}{ccc} \left[\begin{array}{ccc} a_{TX}^{\mathrm{ULA}}\left(\phi_{1}\right) & \cdots & a_{TX}^{\mathrm{ULA}}\left(\phi_{G_{TX}}\right) \end{array}\right] & & \text{for ULA}\\ {}[\begin{array}{cccc} a_{TX}^{\mathrm{UPA}}\left(\phi_{1},\theta_{1}\right) & \cdots & a_{TX}^{\mathrm{UPA}}\left(\phi_{G_{TX}^{\phi }},\theta_{1}\right) & \cdots \end{array} & & \text{for UPA}\\ \begin{array}{ccc} a_{TX}^{\mathrm{UPA}}\left(\phi_{1},\theta_{G_{TX}^{\theta }}\right) & \cdots & a_{TX}^{\mathrm{UPA}}\left(\phi_{G_{TX}^{\phi }},\theta_{G_{TX}^{\theta }}\right) \end{array}] & & \end{array}\right.\\ A_{RXD}=\left\{ \begin{array}{ccc} \begin{array}{c} \left[\begin{array}{ccc} a_{RX}^{\mathrm{ULA}}\left(\phi_{1}\right) & \cdots & a_{RX}^{\mathrm{ULA}}\left(\phi_{G_{RX}}\right) \end{array}\right] \end{array} & & \text{for ULA}\\ \begin{array}{c} {}[\begin{array}{cccc} a_{RX}^{\mathrm{UPA}}\left(\alpha_{1},\varphi_{1}\right) & \cdots & a_{RX}^{\mathrm{UPA}}\left(\alpha_{G_{RX}^{\alpha }},\varphi_{1}\right) & \cdots \end{array} \end{array} & & \text{for UPA}\\ \begin{array}{ccc} a_{RX}^{\mathrm{UPA}}\left(\alpha_{1},\varphi_{G_{RX}^{\varphi }}\right) & \cdots & a_{RX}^{\mathrm{UPA}}\left(\alpha_{G_{RX}^{\alpha }},\varphi_{G_{RX}^{\varphi }}\right) \end{array}] & & \end{array}\right. \end{array}$$

where $A_{TXD}\in {\mathbb{C}}^{N_{TX}\times G_{TX}}$ and $A_{RXD}\in {\mathbb{C}}^{N_{RX}\times G_{RX}}$ are defined for ULA. Here, $G_{TX}$ and $G_{RX}$ denote uniform fine grid points in [−π/2,π/2) at the BS and MS, respectively. Also, $A_{TXD}\in {\mathbb{C}}^{N_{TX}\times \left(G_{TX}^{\phi }\times G_{TX}^{\theta }\right)}$ and $A_{RXD}\in {\mathbb{C}}^{N_{RX}\times \left(G_{RX}^{\phi }\times G_{RX}^{\theta }\right)}$ are defined for the UPA. Here, $G_{TX}^{\phi }\left(G_{TX}^{\theta }\right)$ and $G_{RX}^{\alpha }\left(G_{RX}^{\varphi }\right)$ denote uniform fine grid points in the azimuth (elevation) domain with [−π/2,π/2) ([0,π)) at the BS and MS, respectively. In Equation (17), $A^{\bar{c}}$ is an $N_{B}N_{I}\times G_{TX}G_{RX}$ and $N_{B}N_{I}\times \left(G_{RX}^{\phi }\times G_{RX}^{\theta }\times G_{TX}^{\phi }\times G_{TX}^{\theta }\right)$ matrix for ULA and UPA, respectively. In Equation (2), it is assumed that azimuth (elevation) AoA and AoD, $\alpha_{ml}^{\bar{c}}\left(\varphi_{ml}^{\bar{c}}\right)$ and $\phi_{ml}^{\bar{c}}\left(\theta_{ml}^{\bar{c}}\right)$, are taken from the above-mentioned uniform fine grids. Next, all the $N_{B}N_{I}$ correlation values are stacked in a vector form as follows:

(19) $$Z^{\bar{c}}=\left[\begin{array}{cccc} Z^{\bar{c},0} & Z^{\bar{c},1} & \ldots & Z^{\bar{c},N_{b}-1} \end{array}\right]$$

(20) $$Z^{\bar{c},b}=\left[Z_{0}^{\bar{c},b}\;Z_{1}^{\bar{c},b}\;\ldots \;Z_{N_{i}-1}^{\bar{c},b}\right]$$

Equations (17) and (19) are used to obtain AoAs and AoDs of channel paths. The orthogonal matching pursuit (OMP) algorithm is used to obtain these values because of its simplicity and fast implementation [23,28]. Stagewise OMP (StOMP) or generalized OMP (gOMP) algorithms can also be considered for estimation of AoAs and AoDs of channel paths [29,30]. Using the vector $a^{\bar{c}}$ obtained by OMP, comprised of indices of AoAs and AoDs for all $N_{M}N_{L}$ propagation paths, multipath gains $\beta^{\bar{c}}$ can be estimated as follows:

(21) $${\tilde{\beta }}^{\bar{c}}={\left({A^{\bar{c}}}^{\prime }(a^{\bar{c}})A^{\bar{c}}(a^{\bar{c}})\right)}^{-1}{A^{\bar{c}}}^{\prime }(a^{\bar{c}})Z^{\bar{c}}$$

Then, the estimated channel matrix can be constructed for each candidate cell as follows:

(22) $${\tilde{H}}^{\bar{c}}=A_{RXD}diag\left({\tilde{\beta }}^{\bar{c}}\right){A^{\prime }}_{TXD}$$

Finally, a serving cell should be selected in the initialization stage, taking into consideration the hybrid beamforming structure, because a hybrid beamforming system will be used in the data transmission stage. Here, we select the serving cell that maximizes channel capacity in the hybrid beamforming system. The unconstrained optimal precoder $F_{\mathrm{opt}}^{\bar{c}}$ for hybrid beamformer is obtained by performing a singular value decomposition (SVD) of the estimated channel in Equation (22) as ${\tilde{H}}^{\bar{c}}=U^{\bar{c}}\Sigma^{\bar{c}}V^{\bar{c}*}$ [13]. Here, $U^{\bar{c}}$ is an $N_{RX}\times N_{RX}$ unitary matrix, $\Sigma^{\bar{c}}$ is an $N_{RX}\times N_{TX}$ diagonal matrix, and $V^{\bar{c}}$ is an $N_{TX}\times N_{TX}$ unitary matrix. The matrix $V^{\bar{c}}$ can be expressed as:

(23) $$V^{\bar{c}}=\left[\begin{array}{cccc} V_{1}^{\bar{c}} & V_{2}^{\bar{c}} & \ldots & V_{N_{TX}}^{\bar{c}} \end{array}\right]$$

where $V_{N_{TX}}^{\bar{c}}={\left[\begin{array}{cccc} V_{N_{TX},1}^{\bar{c}} & V_{N_{TX},2}^{\bar{c}} & \ldots & V_{N_{TX},N_{TX}}^{\bar{c}} \end{array}\right]}^{T}\in {\mathbb{C}}^{N_{TX}\times 1}$.

The optimal unconstrained precoder for ${\tilde{H}}^{\bar{c}}$ is given by $F_{\mathrm{opt}}^{\bar{c}}=V_{1}^{\bar{c}}$. In general, the channel capacity of a MIMO system is expressed in terms of the channel matrix ${\tilde{H}}^{\bar{c}}$ and precoder $F_{\mathrm{opt}}^{\bar{c}}$, as follows [31]:

(24) $$R^{\bar{c}}=\log_{2}\left|I_{N_{s}}+\frac{\mathrm{SNR}}{N_{s}}{\tilde{H}}^{\bar{c}}F_{\mathrm{opt}}^{\bar{c}}\times {\left(F_{\mathrm{opt}}^{\bar{c}}{\tilde{H}}^{\bar{c}}\right)}^{\prime }\right|$$

When $N_{RF}$ Tx chains are available in the hybrid beamformer at the BS, Equation (24) can be rewritten as:

(25) $$R^{\bar{c}}=\log_{2}\left|I_{N_{s}}+\frac{\mathrm{SNR}}{N_{s}}\Sigma_{N_{s}}^{\bar{c}}{V_{N_{s}}^{\bar{c}}}^{\prime }F_{\mathrm{opt}}^{\bar{c}}\times {\left(\Sigma_{N_{s}}^{\bar{c}}{V_{N_{s}}^{\bar{c}}}^{\prime }F_{\mathrm{opt}}^{\bar{c}}\right)}^{\prime }\right|$$

where $\Sigma_{N_{s}}^{\bar{c}}=\left[\begin{array}{ccc} \sum_{1}^{\bar{c}} & \cdots & 0\\ \vdots & \ddots & \vdots \\ 0 & \cdots & \sum_{N_{RF}}^{\bar{c}} \end{array}\right]\;\mathrm{and}\;V_{N_{s}}^{\bar{c}}=\left[\begin{array}{ccc} V_{1}^{\bar{c}} & \cdots & V_{N_{RF}}^{\bar{c}} \end{array}\right],$$N_{s}=N_{RF},\;N_{RF}\in N_{TX}\;\mathrm{and}\;N_{RF}\leq N_{TX}$.

By repeating the same procedure for all candidate cells, the cell providing the maximum channel capacity is selected as the optimal serving cell as follows:

(26) $$\mathrm{Serving}\;\mathrm{Cell}\left(\hat{c}\right)=\underset{\bar{c}}{\arg\max}\left(R^{\bar{c}}\right)$$

After the serving cell is selected, the estimated vectors of AoDs (${\hat{\phi }}^{\hat{c}}$) and AoAs (${\hat{\alpha }}^{\hat{c}}$), corresponding to the indices of the array response vector, in the azimuth plane are given as follows:

(27) $$\begin{array}{l} {\hat{\phi }}^{\hat{c}}=\frac{a^{\hat{c}}}{G_{TX}^{\phi }-1}\\ {\hat{\alpha }}^{\hat{c}}=G_{RX}^{\alpha }1_{1\times N_{RF}}-{\hat{\phi }}^{\hat{c}} \end{array}$$

where $1_{1\times N_{RF}}$ is a $1\times N_{RF}$ vector with all values of 1.

## 4. Simulation

**Figure 3:**
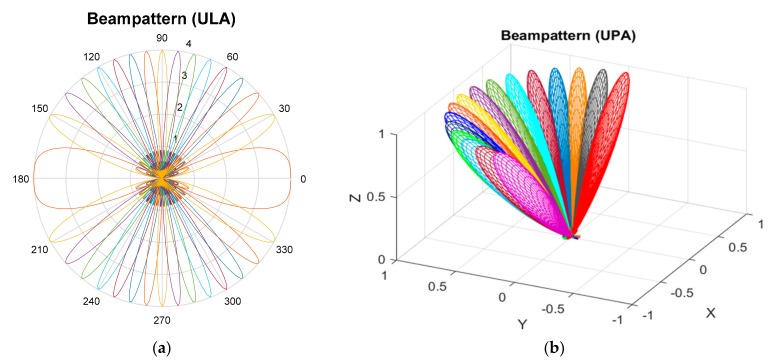
Beampattern of unitary discrete Fourier transform (DFT) matrix $\left(N_{B}=16\right)$, (**a**) uniform linear array (ULA); (**b**) uniform planar array (UPA).

In this section, the performance of the proposed cell selection technique is evaluated by computer simulation in a simple scenario shown in Figure 1. Here, an MS is located at the cell boundary between two cells (CID = 0 with root index = 54, and CID = 1 with root index = 59). The system bandwidth, carrier frequency, and fast Fourier transform (FFT) size are set to 500 MHz, 28 GHz, and 2048, respectively [9,18,32]. For ULA, the number of antenna elements is set to 16 and 8 in the BS and MS, respectively. For UPA, the number of antenna elements is set to 64 $\left(N_{e}=N_{f}=8\right)$ and 16 $\left(N_{e}=N_{f}=4\right)$ in the BS and MS, respectively. In addition, $N_{B}=16$ and $N_{I}=8$ for both ULA and UPA. The length of CBSS sequence, $N_{z}$, is set to 1021. It is assumed that an LoS path exists in both cells. In addition to the LoS path, there exist NLoS paths in cell 0 and the number of NLoS paths (clusters) is set to 4 [33]. For simulation, the spatial channel model (SCM) is used with a *k*-factor of 6 dB [34]. The number of rays in the NLoS path in a Rician fading channel is set to 20 $\left(N_{L}=20\right)$. In Figure 3, beampatterns of unitary DFT matrices with $N_{B}=16$, used in the simulation, are shown for ULA and UPA. Here, $G_{TX}=G_{TX}^{\phi }=180$ and $G_{TX}^{\theta }=1$. The fine grid point is increased with 1 degree of resolution in azimuth angle. For UPA, the elevation angle is fixed to π/4 for convenience.

**Figure 4:**
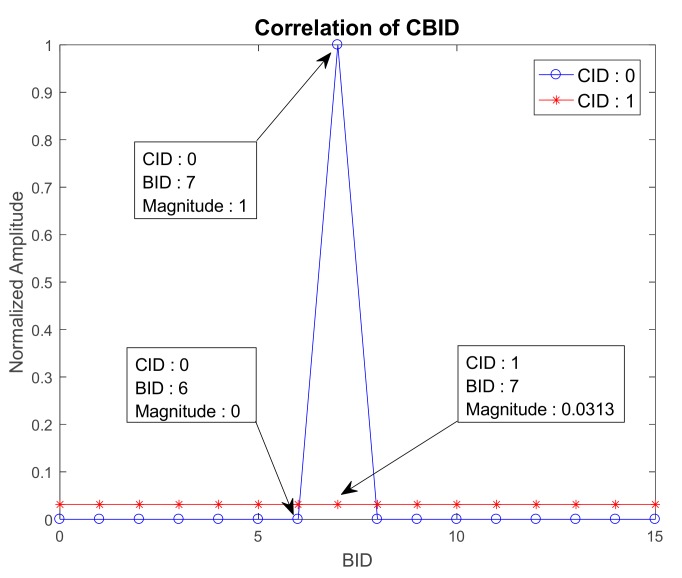
Correlation property of cell and beam synchronization signal (CBSS) when symbol timing offset (STO) does not exist.

**Figure 5:**
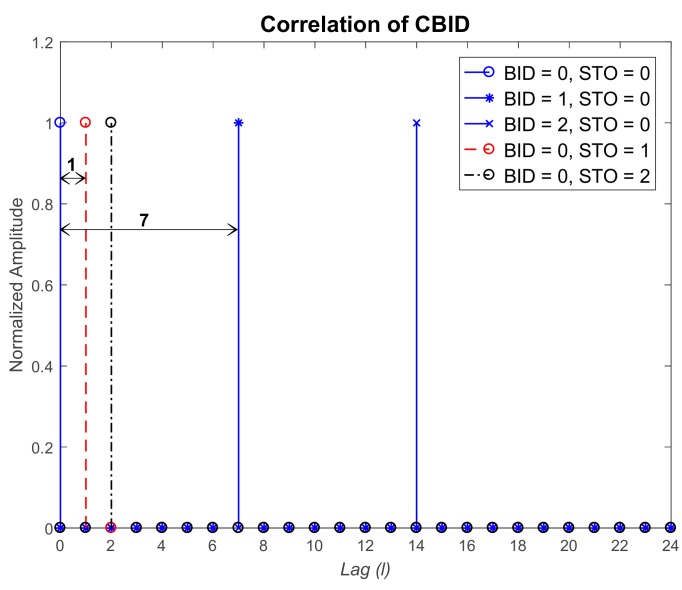
Correlation property of CBSS when STO exists.

Before applying the proposed technique to the mm-wave cellular system, the properties of the proposed CBSS, discussed in Section 3, are verified. In Figure 4, the correlation property of the CBSS is shown when STO is not present. Here, the reference CID and BID are set to 0 $\left(r^{\bar{c}}=54\right)$ and 7, respectively. The parameter *L* is set to 1. From Figure 4, it can be seen that the intra-beam correlation in the same cell (CID = 0) is zero except in the case of the same BID (BID = 7), as given by Equation (9). The normalized inter-cell correlation with the CID equal to 1 $\left(r^{\bar{c}}=59\right)$ is equal to 0.03 $\left(1/\sqrt{N_{z}}\right)$ for all values of BID, as given by Equation (10). Figure 5 shows the correlation property of the CBSS in the time domain when STO exists. The reference CID and BID are set to 0 $\left(r^{\bar{c}}=54\right)$ and 0, respectively. It can be seen that when the STO is zero, correlation values are zero except for the lag *l* = 0. When the values of STO are 1 and 2, the peak shifts by one and two lags, respectively. So, the shift increases proportionally to the STO value. If the parameter *L* is set to 1, there may be an error in BID detection. Therefore, the parameter *L* satisfying Equation (13) should not be selected when the CBSS is designed. To avoid misdetection in the channel environment $\left(\delta_{t_{\max}}=6\right)$, the parameter *L* is set to 7 in the simulation. Figure 5 shows the correlation results when the parameter is used. Peaks can be found at 7 and 14, when CBSSs with BIDs equal to 1 and 2 are used, respectively.

**Figure 6:**
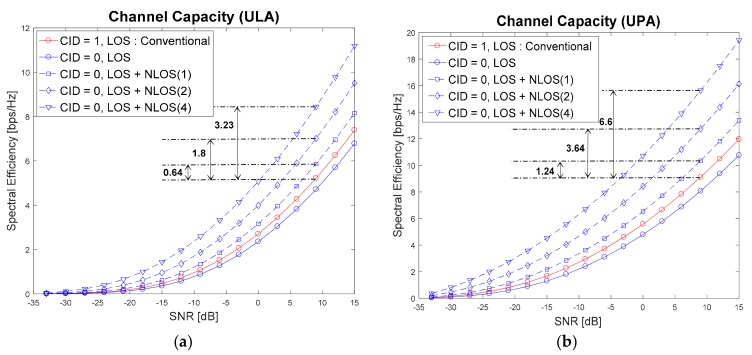
Achievable channel capacity when the conventional and proposed techniques are used.

Figure 6 shows the achievable channel capacity when the conventional and proposed techniques are used. In the conventional technique, the serving cell is selected by measuring SNRs for all candidate cells with Tx/Rx beam sweeping, and choosing the cell with the highest SNR. Cell 1 is selected as the serving cell in the conventional technique because cell 1 has a stronger LoS path than cell 0. However, in the proposed technique (LoS + NLoS(1), LoS + NLoS(2), LoS + NLoS(4)), cell 0 will be selected as the serving cell. That is because more multipath channels are considered for cell selection in the proposed technique as the number of RF chains $\left(N_{RF}\right)$ increases. When $N_{RF}$ is set to {2, 3, 5}, an additional gain of {0.64, 1.8, 3.23} and {1.24, 3.64, 6.6} bps/Hz at an SNR of 9 dB can be achieved for ULA and UPA, respectively. In the proposed technique, the serving cell is selected using Equations (25) and (26).

**Figure 7:**
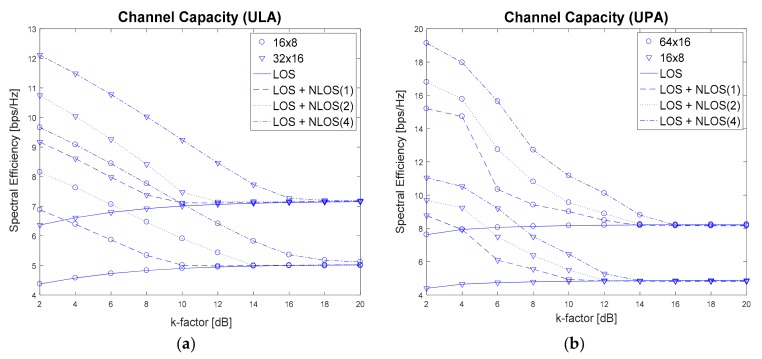
Achievable channel capacity when the proposed technique is used for varying *k*-factor and different antenna configurations.

Figure 7 shows the achievable channel capacity when the proposed technique is used with varying *k*-factor and different antenna configurations. Figure 7a shows the results for ULA with 16 × 8 and 32 × 16 antenna configurations and Figure 7b shows the results for UPA with 64 × 16 and 16 × 8 antenna configurations. Here, the channel capacity is measured at SNR 9 dB when *k*-factor varies from 2 to 20 with a step size of 2. As can be seen in Figure 7, spectral efficiencies for all cases (LOS, LOS + NLOS(1), LOS + NLOS(2), LOS + NLOS(4)) converge towards LOS case, irrespective of ULA/UPA. That is because LOS path becomes stronger than other NLOS paths as *k*-factor increases. The spectral efficiency increases as *k*-factor decreases, because NLOS components become more significant. When the *k*-factor is equal to 6 dB, the ULA configuration with 32 × 16 obtains 2.0–2.5 bps/Hz gain in spectral efficiency over the ULA with 16 × 8, while the UPA configuration with 64 × 16 obtains 4.0–5.5 bps/Hz gain over the UPA with 16 × 8.

**Figure 8:**
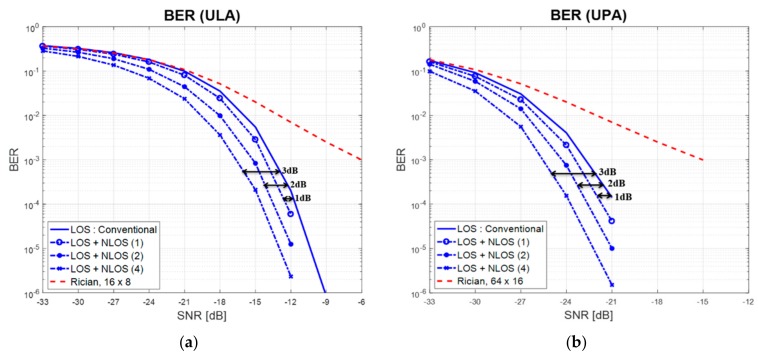
BER performance when the conventional and proposed techniques are used.

Figure 8 shows the bit error rate (BER) performance of the mm-wave system in the data transmission stage after a serving cell is selected by two different techniques. Here, QPSK is used for modulation. For reference, the analytic curve for a Rician fading channel with a *k*-factor equal to 6 dB is inserted. Note that the conventional technique (LoS) is better than the analytic result, because the SCM channel model can be approximated as an AWGN channel in the simulation when the beamforming is performed in the LoS direction. In the analytic result (Rician fading), only the array gain (16 × 8 in ULA, 64 × 16 in UPA) is taken into account. In the conventional technique, BER is measured in cell 1, because cell 1 is selected as the serving cell in the initialization stage. However, the BER is measured in cell 0 in the proposed technique (LoS + NLoS(1), LoS + NLoS(2), LoS + NLoS(4)). As can be seen in Figure 8, an additional maximum-ratio combining (MRC) gain of {1, 2, 3} dB can be achieved in the proposed technique when $N_{RF}$ is set to {2, 3, 5}, respectively.

Table 1 compares the processing time and computational complexity required for channel estimation and cell selection when the conventional and proposed techniques are used. Here, $T_{\mathrm{SS}}$ and $T_{\mathrm{CBSS}}$ represent the lengths of the synchronization signal (SS) and CBSS symbol, respectively. It is assumed that $T_{\mathrm{SS}}$ is equal to the length of two OFDM symbols (PSS and SSS), as in LTE systems, and $T_{\mathrm{CBSS}}$ is equal to one OFDM symbol length. In this example, the number of candidate cells, the number of Tx beams at BS, and the number of Rx beams at MS, are assumed to be $N_{C}=3$, $N_{B}=16$, and $N_{I}=8$, respectively. From Table 1, it can be seen that the processing time required for channel estimation and cell selection with the proposed technique is much smaller than with the conventional technique. In the proposed technique, the processing time decreases proportionally to the number of RF transmit chains. In the conventional technique, the processing time increases linearly with the increase in the number of candidate cells, whereas it does not change in the proposed technique. Next, the computational complexities required for channel estimation are compared. In the conventional technique, CIDs are carried by PSS and SSS, which are transmitted from each beam. Beam sweep is performed at both the BS and MS. However, in the proposed technique, CIDs are carried by PSS and SSS, and BIDs are carried by CBSS in a hierarchical manner. In addition to PSS and SSS, CBSS is also transmitted from each beam simultaneously. Here, beam sweep is performed only at the MS. In the proposed technique, the number of RF transmit chains at the BS is considered to compute computational complexity. Here, N represents a prime number less than minimum bandwidth in LTE and is set to 61. Table 1 shows that, as the number of RF transmit chains increases, the complexity required for channel estimation decreases because multiple beams can be transmitted simultaneously from the BS with minimal interbeam interference, and channel estimation is performed jointly at the MS.

## 5. Conclusions

In this paper, a cell selection technique for mm-wave cellular systems with hybrid beamforming is proposed using the CBSS in the initialization stage. It is shown by simulation that the CID and BID can be detected correctly in a multicell environment with STOs, by assigning an appropriate value to the parameter (*L*) in the CBSS. The proposed technique with the CBSS is shown to require only 6% of the processing time for channel estimation and cell selection, compared to the conventional technique, when the number of RF transmit chains is 8. It is also shown that the proposed cell section technique can achieve 3.64 bps/Hz (UPA) gain in channel capacity or 2 dB gain in BER, when $N_{RF}=3$. Although the cell selection technique is described in this paper for mm-wave cellular systems with hybrid beamforming in the initialization stage, it can also be applied to cell searching in the handover period. 

## Figures and Tables

**Table 1:** Processing time and computational complexity when the conventional and proposed techniques are used.

Factor	Conventional Technique	Proposed Technique	
Processing Time	$T_{\mathrm{SS}}\times N_{I}\times N_{B}\times N_{C}$	$\left(T_{\mathrm{SS}}+T_{\mathrm{CBSS}}\right)\times N_{I}\times \left(N_{B}/N_{RF}\right)$	
Example	3195 μs	$N_{RF}=1$	1597 μs
		$N_{RF}=2$	799 μs
		$N_{RF}=8$	200 μs
Number of complex multiplications	PSS + SSS $\left(62\left(504/3+4\right)\times N_{B}N_{I}\right)$	PSS + SSS + CBSS $\left(\left(62\left(504/3+4\right)+N+\left\lceil N\log_{2}N/2\right\rceil \right)\times N_{B}N_{I}/N_{RF}\right)$	
Example	1,364,992	$N_{RF}=1$	1,411,328
		$N_{RF}=2$	705,664
		$N_{RF}=8$	176,416
